# Integrative eQTL and Mendelian Randomization Analyses with Experimental Validation Prioritize Genetic Candidate Biomarkers for Crohn’s Disease

**DOI:** 10.2174/0118715303433720260216212725

**Published:** 2026-04-21

**Authors:** Xinwei Zhang, Lin Liu, Wenyu Xu, Guangxin Li, Qiu Qin, Jinan Zhang

**Affiliations:** 1 Graduate School, Shanghai University of Traditional Chinese Medicine, Shanghai 201203, People’s Republic of China;; 2 Department of Endocrinology and Rheumatology, Shanghai University of Medicine & Health Sciences Affiliated Zhoupu Hospital, Shanghai 201318, People’s Republic of China;; 3 Department of Endocrinology, Nanyang Central Hospital, Henan 473005, People’s Republic of China;; 4 Shanghai University of Traditional Chinese Medicine, Shanghai 201203, People’s Republic of China

**Keywords:** Crohn’s disease, Mendelian randomization, transcriptomics, gene signature, immune characterization, eQTL anaylysis

## Abstract

**Introduction:**

Crohn's disease (CD) is a chronic, idiopathic inflammatory bowel disease. Understanding the genetic and immunological basis of CD can lead to better therapeutic strategies.

**Methods:**

We employed publicly available datasets from the Gene Expression Omnibus (GEO) database to identify instrumental variables through expression quantitative trait loci (eQTL) analysis and conducted two-sample Mendelian randomization (MR) analyses. Additionally, we investigated the functions and pathways of co-expressed genes using GO, KEGG, and GSEA analyses. The association between these genes and immune cells was examined through immune cell infiltration assessment. Finally, the co-expressed genes were validated.

**Results:**

Several genes, including BATF2, SERPINB9, FCGR2A, MCTP1, SNX30, and SMIM1, were identified as having a causal relationship with CD. These genes are involved in important biological processes and pathways, including autoimmune regulation and oxidative stress. Moreover, CIBERSORT analysis demonstrated a unique distribution of immune cells in CD. Monocytes were significantly decreased in the disease group’s tissues. Immune cell infiltration analysis further emphasized the distinct immune landscapes within this disease.

**Discussion:**

This study identified key genes associated with Crohn's disease, involving immune regulation and other factors, supplementing existing research. Despite limitations such as reliance on public data and insufficient experimental validation, the results provide a basis for exploring potential targets and conducting in-depth mechanism studies.

**Conclusion:**

The results of our study on the molecular mechanism of CD offer new insights into the identification of key genetic traits and potential therapeutic targets, which will enhance our understanding of the disease and lead to the development of more effective treatment options.

## 1 INTRODUCTION

Crohn's disease (CD) is a complex autoimmune disease, which belongs to one of the inflammatory bowel diseases along with ulcerative colitis (UC). The disease mainly occurs in the terminal ileum and adjacent colon. The main clinical manifestations are abdominal pain, diarrhea, and weight loss, often accompanied by local fistulas, perianal lesions, and even various parenteral symptoms and systemic complications [1]. Its pathogenic factors are complex and diverse, and its pathogenesis is still unclear. The pathogenesis of autoimmune diseases is primarily attributed to immune system dysregulation, alterations in the gut microbiota, genetic predisposition, and various environmental factors [2, 3]. Laboratory tests for fecal calprotectin, endoscopy, and imaging are important tools for the diagnosis and monitoring of CD [1]. The incidence of Crohn's disease is different in different areas, but the overall trend is upward. The prevalence of CD is higher in European and American countries, even exceeding the prevalence of UC. Nevertheless, in recent years, the incidence of CD in Western developed countries have stabilized, while the incidence of CD in newly industrialized countries of Asia, Africa, and South America continues to increase [4]. The prevalence is increasing, especially in adults and children, and is common in people in their mid-20s [5]. The specific sex distribution of the disease is rarely found in Western countries, but in some parts of South Korea, men have about three times the risk of women [6].

At present, the main treatment methods of CD include the use of 5-aminosalicylic acid, glucocorticoids, immunomodulatory drugs, antibiotics, and biological agents [7, 8]. However, although these treatments can effectively control the symptoms and reduce the recurrence, there may be adverse reactions, and there is still no effective cure for CD. Among the factors leading to the development of CD, genetic susceptibility is at the top of the list, and studies have shown that people with CD often have specific genetic variants that may affect the function of the immune system, making it more likely to overreact to gut bacteria or other microorganisms [9, 10]. At present, the pathogenesis of CD is still unclear, so the work of exploring the key pathogenic genes and protective genes is particularly important for our specific understanding and treatment of CD. The development of high-throughput sequencing technology allows us to obtain a large amount of genome sequence data quickly and accurately, facilitating a deeper understanding of the mechanisms underlying the occurrence of diseases and contributing to the early prediction, diagnosis, and treatment of the disease.

Mendelian Randomization (MR), as a causal inference method using genetic variables as a tool, can effectively avoid the traditional confounders and common problems in observational studies. Genome-wide association studies (GWAS) identify single-nucleotide polymorphisms (SNPs) linked to specific diseases [11]. Genetic variants associated with diseases or traits can be found by association analysis of genotypes and phenotypes in large-scale samples. Combining GWAS data with eQTL data for analysis allows the identification of genes whose expression levels are associated with complex traits due to pleiotropy [12]. We will conduct MR Analysis to obtain disease-causing genes related to CD, KEGG, and GO analysis of key genes, and GSEA analysis and evaluation of immune infiltration, hoping to clarify the molecular biological mechanism of CD and obtain potential therapeutic targets through the above multi-angle analysis.

## 2 MATERIALS AND METHODS

### 2.1 Data Collection Strategy

Three datasets on Crohn's disease were obtained from the Gene Expression Omnibus (GEO) database (https://www.ncbi.nlm.nih.gov/geo/) hosted by the National Center for Biotechnology Information (NCBI), were GSE16879, GSE36807, and GSE112366, of which the first two is used as the experimental set, the latter data set with a larger sample size was used as the validation set (Table **1**). The selection criteria are: (1) Experiment type is Expression profiling by array. (2) Organism is Homo sapiens, and the tissue is small intestine tissue. (3) The two experimental groups come from the same platform, while the validation set should be from a different platform. (4) The total number of participants in each dataset is greater than or equal to 20. (5) Each dataset includes both a CD disease group and a control group. The GSE16879 dataset includes biopsy samples taken from active inflammatory sites at a certain distance from the ulcers, with 37 CD patients (19 colon and 18 ileum) from a total of 61 IBD patients, and 12 control subjects undergoing polyp screening (6 colon and 6 ileum). The GSE36807 dataset includes 13 CD patients with colon samples, whose disease activity was assessed based on abdominal pain, stool consistency and frequency, systemic symptoms, and 7 healthy control subjects. Both GSE16879 and GSE36807 come from the same platform, GPL570. The validation set is selected from the GSE112366 dataset, which includes ileal biopsies from 26 healthy subjects and 141 baseline untreated CD patients.

### 2.2 Detection of Differential Expression Genes (DEGs)

Combined with GEO data, PCA analysis was performed to standardize the expression values of each gene. The ggplot2 and “ggpubr” packages of the R package were used to draw principal component score maps and to visualize the relationship between samples. Significantly differentially expressed genes were obtained by setting the logFC filtered difference multiple to 1.5 times and the corrected *p* value < 0.05. We utilized the “limma” package in R software to analyze the differentially expressed genes between the case group and the healthy control group. The “heatmap” package and “ggVolcano” were used to visualize the results. The results are shown by cluster heat map and volcano map.

### 2.3 Selection of Instrumental Variables for Analysis

Exposure data extracted from GWAS of summary statistics (https://gwas.mrcieu.ac.uk/) of the eQTL dataset. To investigate the genetic architecture of gene expression in blood and understand the genetic basis of complex traits, a large-scale genome-wide meta-analysis of 19,942 genes in blood was performed [13]. In the present study, genetic instrument variables were selected using stringent criteria: variants were required to be strongly associated with the expression of the corresponding gene at genome-wide significance (*p* < 5×10^-8^). In addition, to reduce redundancy due to linkage disequilibrium, clumping was performed with a window size of 10,000 kb and an LD threshold of r^2^ < 0.001 for SNP selection.

### 2.4 Collection of Outcome Data

This study utilized a CD database consisting of genome-wide association study (GWAS) data selected from the FinnGen dataset (https://www.finngen.fi/en/) named finngen_R10_K11_CD_STRICT2. It includes 16,380,466 SNPs, with 848 cases and 217,852 controls. Both sexes were included, the observational population was European, and the data used were obtained from public databases and can be downloaded freely. This study was a secondary analysis of the data.

### 2.5 Study Design

#### 2.5.1 Mendelian Randomization Analysis

In Mendelian randomization analysis, the inverse- variance weighting (IVW) method is employed as a core analysis method. This method entails weighting the instrumental variable estimates in order to calculate the total effect of causation. In the IVW method, we adopted a fixed effect model based on the assumption of minimal heterogeneity across instrumental variables. Given the consistency in genetic instrument effects and the homogeneity of the study population, a fixed effect model was deemed appropriate for estimating a consistent causal effect. Sensitivity analyses further supported this choice, as results were consistent across both fixed and random effect estimates. To improve transparency and robustness, multiple complementary sensitivity analyses were conducted, including the weighted median, MR-Egger, weighted mode, and simple mode methods. Directionality was evaluated using Steiger filtering/testing to verify that the genetic instruments explained more variance in the exposure than in the outcome, supporting the assumed exposure→outcome direction. Horizontal pleiotropy was assessed using the MR-Egger intercept test and additionally using MR-PRESSO (global test). When MR-PRESSO identified outliers, the outlier-corrected estimates were reported, and the distortion test was used to evaluate whether outlier removal materially changed the causal estimate.

To assess the validity of our instrumental variables, we calculated the F-statistics for each SNP used in the MR analysis. F-statistics were derived from the square of the SNP-exposure effect estimate divided by its standard error squared, providing a measure of instrument strength. An F-value greater than 10 was considered indicative of a strong and valid instrument. Power calculations were performed to ensure that our study was adequately powered to detect the hypothesized causal effects, assuming various effect sizes and prevalences of exposure.

In addition, to quantify the heterogeneity among instrumental variables, we used Cochran's Q statistic, which indicates that there was no significant heterogeneity in the studies when the *p*-value of the Q statistic was greater than 0.05. Finally, the “leave-one-out test” was used to exclude each instrumental variable one by one to evaluate the changes in MR Results after removing any instrumental variable, and to further verify the stability and reliability of the results. The application of these comprehensive analysis methods can improve the reliability of causal inference and is expected to improve the accuracy and scientific rigor of research conclusions.

#### 2.5.2 Functional Enrichment *via* GO and KEGG

The differential genes were taken to intersect with the genes after Mendelian randomization analysis, the up-regulated genes were taken to intersect with the high-risk genes, and the down-regulated genes were taken to intersect with the low-risk genes, the two intersecting genes were obtained, and the results were presented in a Venn diagram. The data were filtered according to a *p*-value less than 0.05, and GO analysis was performed using the “circlize” package to study the roles of intersection genes in three aspects: biological process, cell component, and molecular function. At the same time, KEGG analysis was performed to obtain the pathways through which the intersection genes mainly affected the disease.

#### 2.5.3 GSEA Enrichment Analysis

GSEA (Gene Set Enrichment Analysis) is used to analyze gene expression data to determine how enriched a particular gene set is under different conditions. With GSEA, it is possible to observe which biological pathways are activated in the high and low-expression groups of the target gene. This approach not only helps to identify pathways that are significantly altered under specific conditions but also reveals the role of these pathways in disease occurrence and progression, leading to the discovery of potential therapeutic targets.

#### 2.5.4 Immune Cell Infiltration Analysis

The abundance of immune cells is estimated from complex intestinal tissue expression data by bioinformatics analysis methods such as “CIBERSORT”. The number of different immune cells in the intestinal tissue was estimated in samples from the healthy control (HC), and CD group. This analysis relies on specific gene expression patterns to identify which immune cells exist in samples. The correlation between intersection genes and immune cells was analyzed by means of visual representation in the form of bar and box charts.

#### 2.5.5 Immunohistochemistry (IHC) for Experimental Validation

We selected intestinal tissue samples from 8 patients as the disease group and 8 adjacent normal small-intestinal tissues from trauma cases as controls; all specimens were fixed in formalin, embedded in paraffin, and sectioned onto coated slides (Table **2**). Inclusion criteria for the disease group were: a confirmed diagnosis of Crohn’s disease based on clinical, endoscopic, radiologic, and histologic findings, and availability of archived intestinal FFPE tissue suitable for immunohistochemistry. Control tissues were obtained from trauma surgery cases with histologically normal small intestine. We excluded specimens with insufficient tissue, poor fixation or section quality, or unclear clinical or pathological information. Sections were deparaffinized, rehydrated, and subjected to antigen retrieval using citrate or EDTA buffer. Endogenous peroxidase activity and non-specific binding were blocked. Primary antibodies were incubated with the sections overnight, followed by incubation with secondary antibodies and detection using a streptavidin–HRP system and DAB chromogen. Slides were counterstained with hematoxylin, dehydrated, and mounted for microscopic examination. Among them, the primary antibodies were all purchased from Zen-BioScience and diluted according to the manufacturer's instructions, which were FCGR2A (1:50), SNX30 (1:50), SERPINB9(1:100), and BATF2(1:100). This study was approved by the Ethics Committee of Shanghai Health Medical College (Approval No. 2023-C-123-E01), and written informed consent was obtained from all participants. All procedures were conducted in accordance with the Declaration of Helsinki.

### 2.6 Statistical Analysis

In this study, R software 4.3.0 (http://www.Rproject.org) was used for data processing and analysis. The package “TwoSampleMR” was used for MR and other related analyses, and the package “ggplot2” was used for data visualization. A *P* value below 0.05 was deemed indicative of a significant difference. The immunohistochemical images were quantitatively analyzed using ImageJ software.

## 3 RESULTS

### 3.1 Principal Component Analysis

In this study, two datasets (GSE16879 and GSE36807) were selected for principal component analysis (PCA). Before batch correction, as you can see from Fig. (**1A**), the two data sets on PC1 and PC2 dimension is a clear separation, the separation between samples that is likely to be derived from different experimental batch effects, caused by the systematic differences between different batches of data.

After correcting for batch effects, the two data sets are already basically mixed together in the dimensions of PC1 and PC2, and the separation phenomenon is greatly attenuated (Fig. **1B**). This suggests that the batch effect correction effectively reduced the systematic differences between different batches, making the data more consistent.

### 3.2 DEGs Identification

A total of 21,654 genes from the two datasets were selected for differential gene expression analysis. A total of 939 differentially expressed genes (DEGs) were detected, of which 618 genes were significantly up-regulated, and 321 genes were significantly down-regulated. We employed clustering heat maps (Fig. **2A**) and volcano plots (Fig. **2B**) to visualize differences in gene expression.

### 3.3 MR Analysis

After removal of linkage disequilibrium and elimination of weak variables (F > 10), 26,152 SNPs were obtained as instrumental variables (IVs). Five methods were used for MR Analysis to assess the effect of each SNP on CD. A total of 239 genes associated with CD were identified following a comprehensive screening process, as detailed in the Supplementary Table **S1**.

We intersected the results of differentially expressed genes and MR Analysis, and found that among the down-regulated DEGs, 2 genes also showed an association with reduced disease risk in MR Analysis (OR < 1), and the remaining 319 genes showed down-regulation only in DEG analysis (Fig. **3A**). Among the up-regulated DEGs, four genes also showed an association with increased disease risk in the MR Analysis (OR > 1), and the remaining 614 genes showed upregulation only in the DEG analysis (Fig. **3B**).

We performed MR Analysis of these six genes, and we identified six important genes: BATF2, FCGR2A, MCTP1, SERPINB9, these four genes are significantly up-regulated in the disease group, which are high-risk factors for the occurrence of the disease. SNX30 and SMIM1 are significantly down-regulated in the disease group, which may become protective factors of the disease, showing a significant negative causal relationship with CD.

For BATF2, the Weighted median and IVW analyses yielded *p*-values of 0.021 and 0.013, respectively. The corresponding odds ratios (ORs) were 1.646 (95% CI: 1.077 to 2.517) and 1.634 (1.110 to 2.404).

For FCGR2A, the Weighted median and IVW analyses yielded *p*-values of 0.079 and 0.043, respectively. The corresponding odds ratios (ORs) were 1.374 (95% CI: 0.964 to 1.959) and 1.381 (1.010 to 1.889).

For MCTP1, the Weighted median and IVW analyses yielded *p*-values of 0.033 and 0.033, respectively. The corresponding odds ratios (ORs) were 1.117 (95% CI: 1.009 to 1.237) and 1.112 (1.008 to 1.227).

For SERPINB9, the Weighted median and IVW analyses yielded *p*-values of 0.002 and 0.001, respectively. The corresponding odds ratios (ORs) were 1.250 (95% CI: 1.087 to 1.437) and 1.273 (1.101 to 1.472).

For SMIM1, the Weighted median and IVW analyses yielded *p*-values of 0.045 and 0.028, respectively. The corresponding odds ratios (ORs) were 0.851 (95% CI: 0.727 to 0.996) and 0.858 (0.748 to 0.983).

For SNX30, the Weighted median and IVW analyses yielded *p*-values of <0.001 and 0.005, respectively. The corresponding odds ratios (ORs) were 0.763 (95% CI: 0.655 to 0.887) and 0.772 (0.644 to 0.925).

A forest plot effectively illustrates the causal relationship between specific significantly different genes and the disease (Fig. **3C**). The leave-one-out method and scatter plots were employed to visually present the main findings and sensitivity analyses. We conducted heterogeneity and pleiotropy sensitivity analyses for each co-expressed gene. Directional horizontal pleiotropy was assessed using the MR-Egger intercept test, and the intercept estimate, 95% CI, and *p* value are provided in Supplementary Table **S4**. Overall, MR-Egger intercepts were close to zero, with no evidence of directional horizontal pleiotropy. This implies that the effects of the individual genetic instruments (SNPs) on the exposure are consistent and influence the outcome solely through the exposure, rather than through alternative pathways. These findings bolster the robustness and reliability of our results, further corroborating the causal relationship between exposure and outcome. Furthermore, the consistent outcomes derived from various methods (such as Weighted Median and IVW) underscore the appropriateness of our methodological choices. In summary, the observed effects are predominantly attributable to the exposure, rather than to other potential confounders or systematic biases (Supplementary Figs. **S1** and **2**; Supplementary Tables **S2-S4**). Directionality was assessed using Steiger testing to confirm that the instruments explained more variance in the exposure than in the outcome, supporting the assumed exposure→outcome direction (Supplementary Table **S5**). Heterogeneity across instruments was quantified using Cochran’s Q statistics, with full reporting of Q, degrees of freedom, and *p* values (Supplementary Table **S6**). MR-PRESSO did not identify outlier instruments (n_outliers = 0 across exposures), and global tests were not significant; therefore, outlier-corrected estimates were not applicable (Supplementary Table **S7**).

Additionally, we visualized the intersecting genes using Circos plots to understand their chromosomal distribution (Fig. **3D**). It was found that BATF2 is located on chr11, SERPINB9 on chr6, FCGR2A and SMIM1 both on chr1, MCTP1 on chr5, and SNX30 on chr9.

### 3.4 GO and KEGG Enrichment Analysis

Kyoto Encyclopedia of Genes and Genomes (KEGG) and Gene Ontology (GO) analyses were performed on critical disease genes, collectively revealing the enrichment of significant genes in various biological processes (BP), cellular components (CC), molecular functions (MF), and KEGG pathways. These analyses facilitate a deeper understanding of the potential roles these genes play in cellular and molecular functions and disease mechanisms.

The GO enrichment analysis circular plot indicates that these co-expressed genes are mainly concentrated in MF and BP processes, with fewer in CC (Fig. **4A**). The GO analysis bubble plot reveals that significant genes are enriched in several vital biological processes, including autophagy, lysosomal microautophagy, negative regulation of response to oxidative stress, and natural killer cell-mediated immune response, indicating potential involvement of these genes in these crucial biological processes. Additionally, significant genes are enriched in several critical MF categories, such as IgG binding, cysteine-type endopeptidase inhibitor activity involved in apoptotic process, immunoglobulin binding, and cysteine-type endopeptidase regulator activity involved in apoptotic process, suggesting potential involvement in cellular apoptotic processes. CC might involve the synaptic vesicle membrane, exocytic vesicle membrane, and early endosome membrane (Fig. **4B**).

KEGG analysis reveals that significant genes are enriched in multiple critical pathways, including Leishmaniasis, PD−L1 expression, and PD−1 checkpoint pathway in cancer, Fc gamma R−mediated phagocytosis, and Systemic lupus erythematosus (SLE), suggesting these genes may play vital roles in these pathways. Notably, SLE, an autoimmune disease, is also associated with these genes, implying a potential influence on autoimmune diseases (Fig. **4C**).

### 3.5 GSEA Enrichment Analysis

We conducted GSEA to investigate the enrichment of specific gene sets in high- and low-expression samples. BATF2 express samples, high KEGG signaling pathways such as chemokine signaling pathways, cell factor - cell factor receptor interaction, and other significant enrichment of hematopoietic cell lineage, especially related to immunity and inflammation (Fig. **5A**). In the SERPINB9 high expression samples, significantly enriched KEGG signaling pathways included Leishmania infection, chemokine signaling pathway, cytokine-cytokine receptor interaction, and Toll- like receptor signaling pathway, suggesting that high SERPINB9 expression may enhance immune response and infection defense (Fig. **5B**). KEGG signaling pathways such as chemokine signaling pathway, cytokine-cytokine receptor interaction, and systemic lupus erythematosus were significantly enriched in FCGR2A high expression samples, suggesting that high FCGR2A expression may be related to autoimmunity and inflammatory responses, which is consistent with the enriched KEGG pathways we identified above (Fig. **5C**). Significantly enriched KEGG signaling pathways in MCTP1 high-expression samples included chemokine signaling, cytokine-cytokine receptor interaction, Leishmania infection, and Toll-like receptor signaling. These findings suggest that MCTP1 high expression may affect the regulation of the immune system and cell-to-cell communication (Fig. **5D**). In high SNX30 express samples, KEGG signaling pathways such as butanoate metabolism, citric acid cycle, fatty acid metabolism, peroxidase, and retinol metabolism, and other significant enrichments, prompt SNX30 high expression may be closely related to the biological metabolism and metabolic regulation (Fig. **5E**). In samples with high SMIM1 expression, the significantly enriched KEGG signaling pathways included cell adhesion molecules CAMS, chemokine signaling pathway, cytokine-cytokine receptor interaction, and focal adhesion, suggesting that high SMIM1 expression may affect cell adhesion, migration, and immune responses (Fig. **5F**).

In BATF2 low expression samples, KEGG signaling pathways such as drug metabolism cytochrome p450, metabolism of xenobiotics by cytochrome p450, ppar signaling pathway, retinol metabolism, steroid hormone biosynthesis, etc., were significantly enriched, especially related to metabolic pathways (Fig. **6A**). In the SERPINB9 low expression samples, butanoate metabolism and other KEGG signaling pathways were significantly enriched, indicating that the high expression of SERPINB9 was also related to various biological metabolism (Fig. **6B**). It was also associated with various biological metabolic pathways in FCGR2A low-expression samples (Fig. **6C**). The pathways of cytochrome P450 drug metabolism, other drug-metabolism enzymes, foreign compound metabolism, retinol metabolism, and steroid hormone synthesis all showed negative enrichment in the low-MCTP1 group, indicating a connection with drug metabolism and hormone synthesis (Fig. **6D**). In the low SNX30 group, chemokine signaling, complement and coagulation cascades, cytokine interactions, and systemic lupus erythematosus pathways were associated, which may be related to inflammatory and immune responses, autoimmune diseases (Fig. **6E**). In the low SMIM1 group, it was mainly significantly enriched in drug metabolism and inflammatory response (Fig. **6F**).

In conclusion, we found that chemokine signaling and cytokine-cytokine receptor interactions were active in the high-expression group. It was mainly associated with various biological metabolic pathways in the low-expression group.

### 3.6 Assessment of Immune Cell Infiltration in Crohn’s Disease

Through GO, KEGG pathway, and GSEA, we specifically investigated the infiltration of immune cells in both the healthy control and CD groups. The CIBERSORT algorithm was employed to quantify the proportions of various immune cells within the tissues. CIBERSORT can help identify changes in the proportion of cell types related to specific diseases, and then find potential biomarkers [14]. Some specific cell types, such as naive B cells, memory B cells, plasma cells, T cells, CD8, and monocytes, were different between the control group and the treatment group (Fig. **7A**). We found that the proportion of monocytes was significantly lower in the CD group than in the control group (*p* < 0.05) (Fig. **7B**).

 Correlation analysis revealed a significant negative relationship between mast cells resting and mast cells activated, Macrophages M1 and Plasma cells, Neutrophils and B cells memory, NK cells activated and NK cells resting, Neutrophils and T cells follicular helper. However, Neutrophils show a significant positive correlation with monocytes and activated Dendritic cells. There is also a significant positive correlation between resting Mast cells and resting T cells CD4 memory, and resting Dendritic cells. Additionally, resting Dendritic cells and resting T cells CD4 memory show a significant positive correlation.

Through gene-cell correlation analysis, we found that the intersecting gene BATF2 is positively correlated with T cells CD4 memory activated, Macrophages M1, and Eosinophils. Intersecting gene SERPINB9 is positively correlated with Monocytes, activated Dendritic cells, activated Mast cells, and Neutrophils, and negatively correlated with Plasma cells, resting T cells CD4 memory, resting NK cells, Macrophages M2, resting Dendritic cells, and resting Mast cells. Intersecting gene FCGR2A is positively correlated with Neutrophils, activated Mast cells, activated Dendritic cells, Monocytes, and activated NK cells, and negatively correlated with resting Mast cells, Macrophages M2, resting NK cells, resting T cells CD4 memory, and B cells memory. Intersecting gene MCTP1 is positively correlated with Neutrophils, activated Mast cells, activated Dendritic cells, and Monocytes, and negatively correlated with resting T cells CD4 memory, resting NK cells, resting Dendritic cells, and resting Mast cells. Intersecting gene SNX30 is negatively correlated with Neutrophils and activated Dendritic cells. SMIM1 is negatively correlated with Macrophages M0 and T cells regulatory (Tregs), and positively correlated with activated Dendritic cells, T cells CD4 memory activated, and T cells CD8 (Fig. **7C**).

### 3.7 Validation Group Differential Analysis

We detect whether the intersection genes were different in the validation group (GSE112366), so the experiment was carried out gene expression analysis by boxplot, comparing the Control group (Control) and the CD group (Treat) with six different genes (BATF2, FCGR2A, MCTP1, SERPINB9, SNX30, and SMIM1), and finally 5 genes (BATF2, FCGR2A, MCTP1, SERPINB9, SNX30) were shown. The results showed that the expression of BATF2, FCGR2A, and MCTP1 in CD samples was higher than that in the healthy control group, and the expression of BATF2 was significantly higher in the disease group (*p* <0.001) (Fig. **8**). In addition, SNX30 expression was downregulated in the disease group. SERPINB9 was expressed in healthy control group and the disease was basically unchanged; there was no significant difference. Notably, SMIM1 could not be evaluated in the microarray validation cohort because its expression in intestinal tissues was close to background levels, and despite screening multiple independent datasets, we were unable to identify a cohort with sufficient SMIM1 expression for reliable analysis, so it is not included in the validation results. Obviously, this result was basically consistent with our MR analysis results, which further verified the reliability of our MR analysis results.

### 3.8 IHC Experiments to Validate Different Genes

In this study, immunohistochemistry was used to investigate the differences in the protein expression of BATF2, FCGR2A, SERPINB9, and SNX30 in the small intestine tissues between the Crohn's disease (CD) group and the control (HC) group (Fig. **9A**). The results showed that BATF2 was mainly expressed in the cytoplasm and nucleus of intestinal mucosal epithelial cells. In the healthy control group (HC), BATF2 expression was weak, and only a slight positive signal was observed in the nucleus of some glands. In the Crohn's disease (CD) group, BATF2 expression was significantly enhanced, and the positive staining was concentrated in the nucleus of glandular epithelial cells, especially in the cells around the gland in the area of inflammatory cell infiltration. FCGR2A was mainly localized on the cell membrane of glandular epithelial cells. In the HC group, FCGR2A expression was low, and a sparse positive staining signal was observed only on the cell membrane of a small number of glandular epithelial cells. However, in the CD group, the expression of FCGR2A was significantly enhanced, and strong positive staining was observed in glandular epithelial cells and inflammatory cells, which were mainly distributed in the periglandular and inflammatory infiltration areas. The expression of SERPINB9 was mainly located in the cytoplasm of glandular epithelial cells, and a small amount of SERPINB9 was distributed in the nucleus. In the HC group, the expression of SERPINB9 was low; only slight positive staining was observed in a few glands, and the signal distribution was relatively uniform. In the CD group, the expression of SERPINB9 was significantly increased, and strong positive signals were observed in the cytoplasm of inflammatory areas around the glands and cells in the glands, and positive staining was also observed in the nuclei of some immune cells. However, the expression of SNX30 in the HC group was mainly located in the nucleus, and a positive signal could be seen, while the expression of SNX30 in the CD group was low. Compared with the HC group, the average optical density values of BATF2, FCGR2A, and SERPINB9 positive signals in the CD group were significantly increased (Figs. **9B**-**D**). However, the expression of SNX30 in the CD group was significantly lower than that in the HC group, which was statistically significant (Fig. **9E**).

## 4 DISCUSSION

CD is a chronic non-specific intestinal inflammatory disease and a chronic inflammatory granulomatous disease. Crohn’s disease often has an insidious onset and variable disease course; a subset of patients even has a family history of IBD. Many studies have reported the sequence variation of NOD2, SLC22A4, SLC22A5, DLG5, ABCB1, and other specific genes in CD [15, 16]. More recent work in IBD and immune diseases has combined genetic evidence with transcriptomic data. Using the same general approach, MR evidence was integrated with transcriptomic information to prioritize genes potentially relevant to CD. The resulting candidates require functional validation and may help clarify CD pathogenesis.

In this study, a fixed effect model was selected for the IVW method, based on several considerations. First, the level of heterogeneity across instrumental variables was assessed and found to be minimal, reflecting consistent effects of the genetic variants used. This aligns with the fixed effect model assumption, which presumes a constant causal effect across studies or instruments. Additionally, the homogeneity of the study population and similarity among genetic instruments further justified this choice. By using a fixed effect model. A unified causal estimate was obtained, improving interpretability and precision in the absence of substantial heterogeneity. Sensitivity analyses comparing fixed and random effect estimates also supported the robustness of this model specification.

First, we analyzed three datasets (GSE16879, GSE36807, and GSE112366), of which the first two datasets, belonging to the same platform, were used as the test set, and the remaining one with more samples was used as the validation set. Principal component analysis (PCA) was applied to the test datasets GSE16879 and GSE36807 to correct for batch effects. Subsequently, the two datasets were merged for further analysis. This process identified 939 differentially expressed genes, of which 618 were increased in expression, and 321 were significantly down-regulated. Six key genes were co-expressed, including BATF2, SERPINB9, FCGR2A, MCTP1, SNX30, and SMIM1. The expression of the first four genes was up-regulated, and the expression of the last two genes was down-regulated. We analyzed and visualized the GO function and KEGG pathway of these six genes and found that they are involved in autophagy and apoptosis, oxidative stress response, and inflammatory response. Analysis of GSEA samples revealed that genes with high expression were associated with autoimmune and inflammatory responses, while genes with low expression were associated with drug metabolism and inflammatory response. Using the CIBERSORT algorithm, the specific analysis of gene-immune cells found that monocytes were significantly different between the disease group and the healthy control group, which was consistent with the conclusion of many studies [17]. A prospective longitudinal cohort study found that mononuclear cell count was significantly associated with fecal calcium, protein, and disease recurrence, which is consistent with our findings [18].

BATF2 (Basic leucine zipper ATF-Like transcription factor 2) is a member of the activator protein-1 (AP-1) transcription factor family. Many experiments have shown that CD is Th1-mediated intestinal inflammation, and the expression of Th1 cytokines, including IFN-γ and TNF-α, is increased in CD [19, 20]. In CD, Th1 cells also aggravated intestinal inflammation by inducing macrophage M1 polarization, resulting in increased mucosal barrier permeability, while BATF2 further induced IFN-γ-activated classical macrophage (M1) inflammatory response [19, 21]. Studies indicate that BATF2 can enhance the pro-inflammatory cytokine response in macrophages [22]. BATF2 promotes inflammatory response by regulating the innate immune response and the expression of related genes (such as CXCL9 and CXCL10) in DSS-induced ulcerative colitis mice model [23]. BATF2 is an important regulator of inflammatory response in UC, and its high expression is closely related to the aggravation of inflammation. MANF plays an anti-inflammatory and protective role in the intestinal barrier by negatively regulating the BATF2 signaling pathway [24]. When we performed the validation, we found that this gene was significantly associated with the occurrence of the disease, which was consistent with the conclusion of most studies. Unlike well-known IBD genes such as NOD2 or IL23R, the genes highlighted here (BATF2, *etc*.) point to alternative inflammatory pathways, underscoring the value of our approach.

SERPINB9(Serine protease inhibitor B9) is a serine protease inhibitor that inhibits its protease activity by binding to granzyme B, thereby protecting cells from its induced apoptosis [25]. SERPINB9 also acts as an endogenous caspase-1 inhibitor. Studies have shown that SERPINB9 regulates IL-1β release by inhibiting caspase-1, and identified a serpinB9 variant (A329S) associated with autoinflammatory disease (AID) whose inhibition of caspase-1 is impaired, but whose inhibition of granzyme B is normally retained [26]. SERPINB9, by regulating caspase-1–dependent IL-1β release and cytotoxic pathways, might delineate a subgroup of patients in whom inflammasome activity is particularly relevant, and thus could conceptually intersect with emerging IL-1-targeted or inflammasome-directed therapies, although this remains highly speculative.

FCGR2A (Fc gamma receptor IIA), a type of Fc receptor, specifically binds to the Fc fragment of IgG antibodies. Studies have shown that it is a driver of IBD [27, 28]. There are many polymorphisms in the FCGR2A gene, such as R131H, which are associated with an individual's susceptibility to infections, inflammatory diseases, and autoimmune diseases [29, 30]. In most inflammatory diseases, high expression or functional variants of FCGR2A exacerbate inflammation [31]. The same trend was shown in our study, but in some cases, protective genotypes (such as *519GG or R131) may attenuate the inflammatory response, which is associated with reduced immune complex-mediated cytotoxicity [31]. FCGR2A, as a low-affinity Fcγ receptor on myeloid cells, may influence how patients respond to monoclonal antibody therapies already used in CD (*e.g*., anti-TNF or anti-integrin agents), by modulating Fc-dependent effector functions; if validated, FCGR2A expression or genotype could potentially serve as a biomarker for optimizing antibody choice or dosing.

MCTP1 (Multiple C2 domains, transmembrane 1) is a protein that contains multiple C2 domains and transmembrane domains, and its research is mainly focused on neurological diseases [32]. In the nervous system, MCTP1 is involved in cyclic endocytosis, secretory vesicular recycling, cell migration, and oxidative stress. Abnormalities in MCTP1 may affect cell membrane homeostasis or central nervous system development, leading to neuropsychiatric disorders [33]. At present, there are few studies on this gene in CD. We found that its expression in CD is upregulated, and its cause and result need further study.

Unlike the high expression of the above four genes, BATF2, SERPINB9, FCGR2A, and MCTP1, our study found that SNX30 and SMIM1 were reduced in CD. SNX30 (Sorting Nexin 30) belongs to the sorting connexin family, regulates the formation and transport of endosomes, plays a key role in endocytosis and signal transduction, and its abnormality may be related to various diseases [34, 35]. Studies have shown that the gene expression in lung adenocarcinoma is also reduced [36]. SMIM1 (small integral membrane protein 1) is a tail-anchored transmembrane protein involved in the regulation of erythrocyte production and cell membrane function [37]. SMIM1 expression is increased in hepatocellular carcinoma and decreased in cirrhosis, which may be related to the different levels of maturation of red blood cells in the two diseases [38]. The SMIM1 gene, essential for the determination of red blood cell antigens and influenced by specific cell types, is notably associated with T cell and neutrophil gene clusters in gene co-expression cluster analyses. This association with inflammatory bowel disease (IBD) has been validated, suggesting that SMIM1 may play a significant role in the pathogenesis of IBD [39]. SMIM1’s potential role in IBD, suggested by a co-expression analysis, is reinforced by our MR finding.

Our study found that there was a significant difference in the proportion of monocytes between the CD group and the healthy control group, which was positively correlated with the intersection genes SERPINB9, FCGR2A, and MCTP1. Interestingly, the CIBERSORT analysis suggested a lower proportion of monocytes in CD tissue compared to normal tissue. This could be because many monocytes in the inflamed gut may rapidly differentiate into macrophages or dendritic cells, thus appearing as a reduced monocyte fraction. Despite this, peripheral blood studies show monocyte counts correlate with disease activity, indicating the role of monocyte-lineage cells in CD pathogenesis [18]. Monocytes play an important role in the pathogenesis of a variety of autoimmune diseases because they can differentiate into macrophages and dendritic cells, both of which have important roles in antigen presentation and cytokine production. The above genes can affect the activation and function of monocytes, thereby regulating the immune response. By delving into the specific functions and interactions of these genes in monocytes, we can better understand the complex mechanisms of CD and develop more targeted and effective treatments.

Previous genetics-based studies have established a broad CD genetic architecture, with large GWAS and sequencing efforts identifying numerous susceptibility loci (*e.g*., innate immunity, autophagy, and IL-23/Th17-related pathways) [40-42]. In parallel, multi-omics and transcriptome-wide approaches (*e.g*., TWAS and other integrative frameworks combining GWAS with expression data) have been applied to prioritize candidate genes for IBD/CD, but they differ in study design, tissues, and prioritization logic [43]. Against this background, the present study does not claim that all prioritized genes have never been mentioned in CD. Instead, the added value is the specific integrative workflow and evidence triangulation: genes were first prioritized by intersection with transcriptomic signals and co-expression patterns, then tested using eQTL-based MR as an orthogonal line of genetic evidence, and finally supported using intestinal-tissue expression/validation. For example, FCGR2A has been reported as markedly upregulated in inflamed terminal ileum/colon in CD, consistent with a role in intestinal immune activation [44]. BATF2 has been implicated in regulating macrophage-driven intestinal inflammation in experimental settings [45], and SERPINB9 appears in inflammatory mucosal expression signatures reported in CD/UC [46]. Thus, these results extend existing literature by providing convergent genetic support and prioritization rather than simply re-listing known GWAS genes. In contrast, for candidates with relatively limited CD-focused genetic/transcriptomic evidence (*e.g*., MCTP1, SNX30, SMIM1), the findings should be regarded as exploratory signals that require replication and functional validation.

This study has several limitations. First, MR analyses rely on large-scale biobank resources that are often drawn from specific ethnic or regional groups. The datasets used here were predominantly of European ancestry (including FinnGen), and allele frequencies and effect sizes may differ across ancestries. In addition, ancestry differences between the eQTL source and the outcome GWAS may lead to linkage disequilibrium (LD) mismatches, which can change instrument strength and SNP–outcome associations and thereby bias MR estimates. Second, there is an important tissue mismatch: the MR instruments were derived from blood eQTL, whereas DEGs and experimental validation were based on intestinal tissue. Regulation in blood may not reflect gene regulation in the gut mucosa, particularly in the local inflammatory microenvironment, so tissue-specific effects may be missed, and false positives/negatives are possible. Accordingly, the causal interpretation should be cautious; gut-tissue validation provides supportive evidence for selected candidates but can only partially mitigate this concern. Third, for several co-expressed genes, the number of available cis-eQTL instruments was small, which limits statistical power and constrains sensitivity analyses; therefore, these MR estimates should be considered exploratory. Fourth, the immunohistochemical validation cohort was small (8 CD *vs* 8 controls), so the staining results are supportive rather than definitive and require confirmation in larger, independent tissue cohorts. Finally, SMIM1 was difficult to evaluate in publicly available transcriptomic datasets, with limited studies mainly related to hematologic conditions; despite testing multiple datasets, consistent expression differences between controls and CD were not observed, and further validation with deeper sequencing and functional experiments is warranted.

## CONCLUSION

This study used MR to examine potential causal links between the intersecting genes and CD. Several candidates (BATF2, SERPINB9, FCGR2A, MCTP1, SNX30, and SMIM1) were prioritized as genes of interest that may warrant further investigation as therapeutic or mechanistic targets. While MR can reduce confounding compared with conventional observational designs, important limitations remain. In particular, population structure and ancestry differences between the eQTL source data and the FinnGen outcome dataset may lead to linkage disequilibrium (LD) mismatches and instrument-exposure/outcome association differences, which could bias MR estimates. Therefore, these findings should be interpreted cautiously and validated using ancestry-matched resources and independent cohorts.

## Figures and Tables

**Fig. (1):**
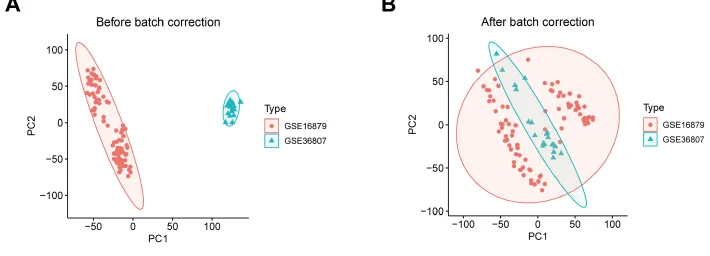
(**A**) Distribution of different datasets in PCA before batch correction. (**B**) Distribution of different datasets in PCA after batch correction. Horizontal axis (PC1) and vertical axis (PC2) represent the first and second principal components. Red circles represent dataset GSE16879, and cyan triangles represent dataset GSE36807.

**Fig. (2):**
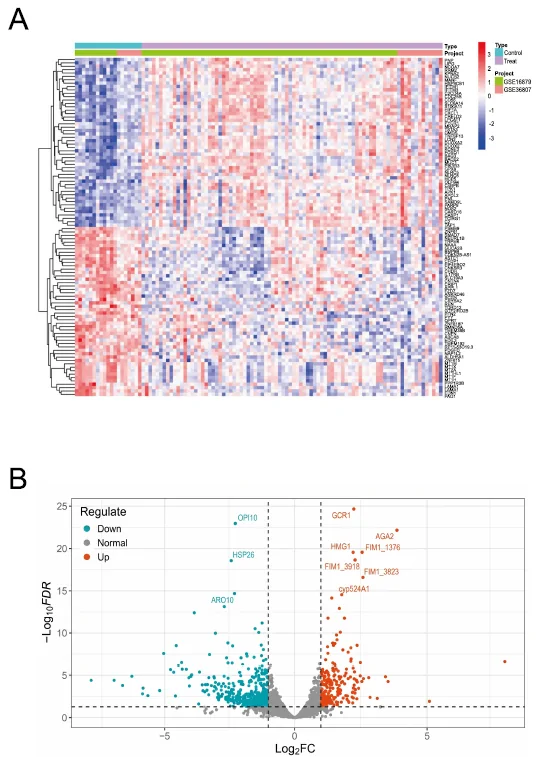
(**A**) Heatmap of the top 50 genes with most significant expression differences. (**B**) Volcano plot of DEGs.

**Fig. (3):**
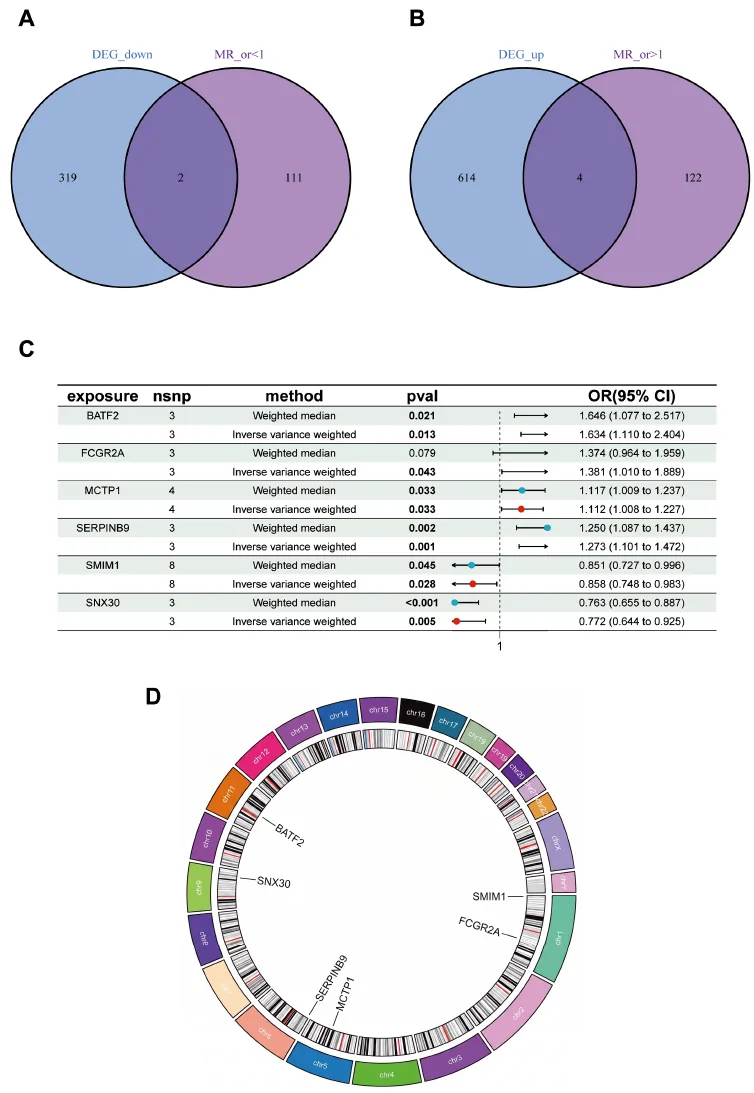
Integrative visualization of DEGs and MR findings in disease association studies. (**A**) Venn plot of downregulated DEGs with MR analysis (OR ≤ 1). (**B**) Venn plot of upregulated DEGs with MR analysis (OR > 1). (**C**) Forest diagram of significant gene-disease associations. (**D**) Visualization of key MR genes in a circos plot.

**Fig. (4):**
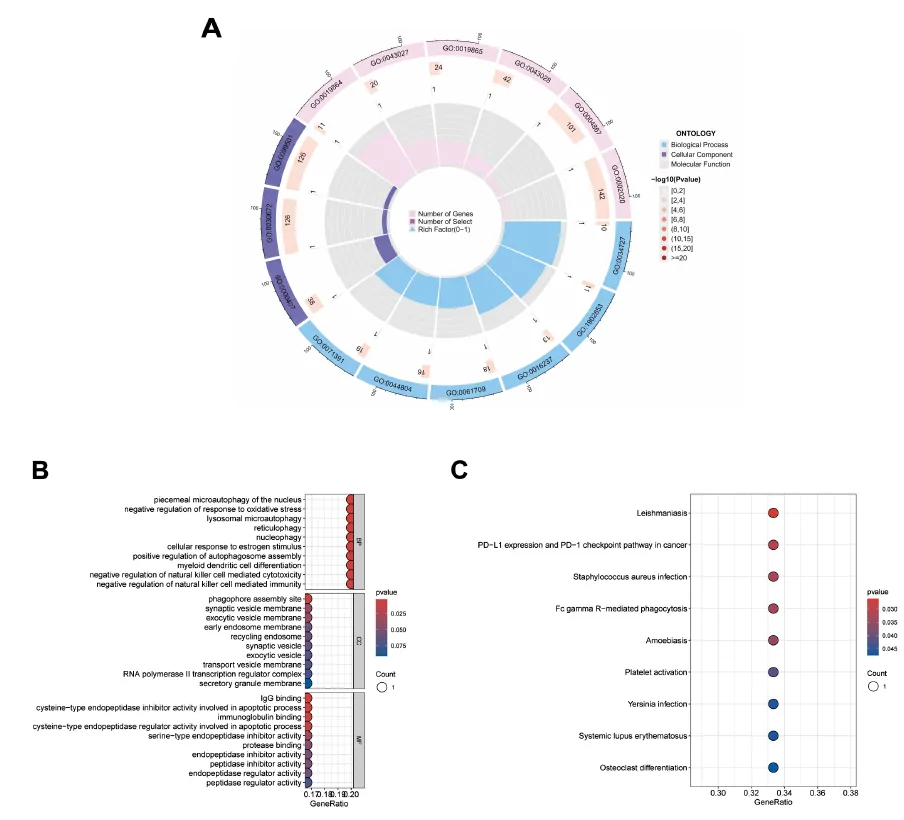
Visualization of significant gene enrichment in gene ontology and KEGG pathways. (**A**) Circular visualization of GO enrichment analysis. Different colors denote distinct GO categories: blue for BP, pink for MF, and purple for CC. Each sector represents a GO term, with internal numbers showing the number of enriched genes. The rich factor indicates the ratio of the number of genes in a given GO term to the number of background genes. (**B**) Bubble plot of GO enrichment analysis. Colors indicate the *p*-value, with red representing more significant enrichment (lower *p*-value) and blue representing less significant enrichment (higher *p*-value). (**C**) Bubble plot of KEGG pathway enrichment analysis. Bubble size indicates the number of genes in the pathway.

**Fig. (5):**
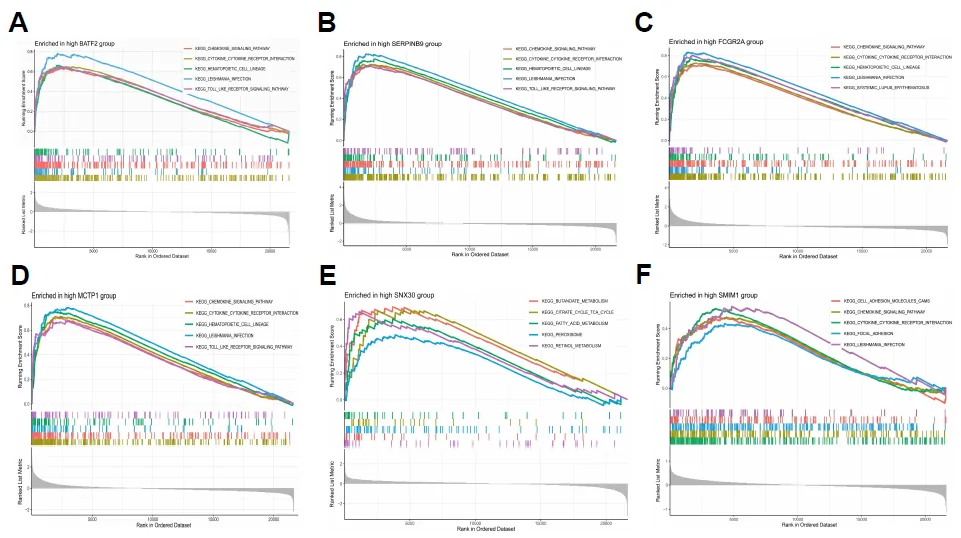
GSEA of high-expression gene groups showing enrichment of different KEGG signaling pathways. (**A-F**) Critical genes: BATF2, SERPINB9, FCGR2A, MCTP1, SNX30, SMIM1.

**Fig. (6):**
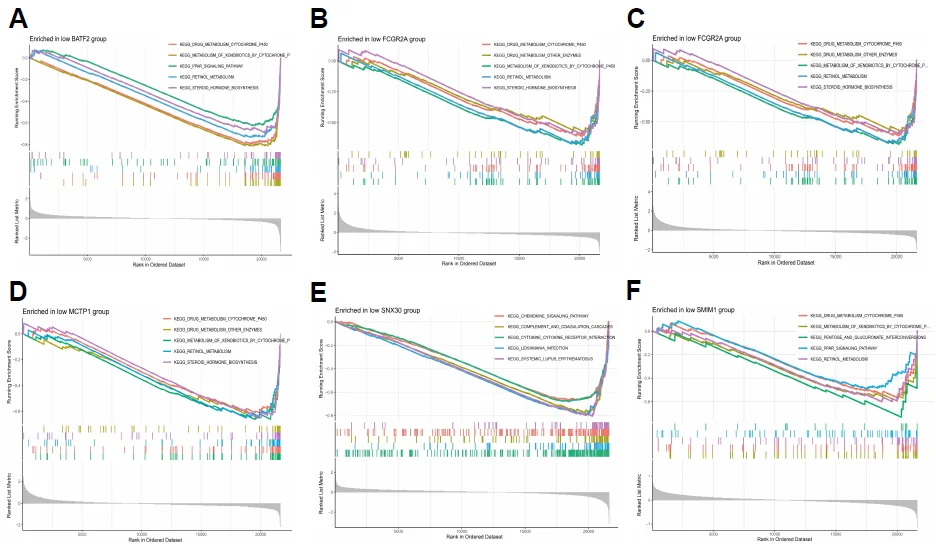
GSEA of low-expression gene groups showing enrichment of different KEGG signaling pathways. (**A-F**) Critical genes: BATF2, SERPINB9, FCGR2A, MCTP1, SNX30, SMIM1.

**Fig. (7):**
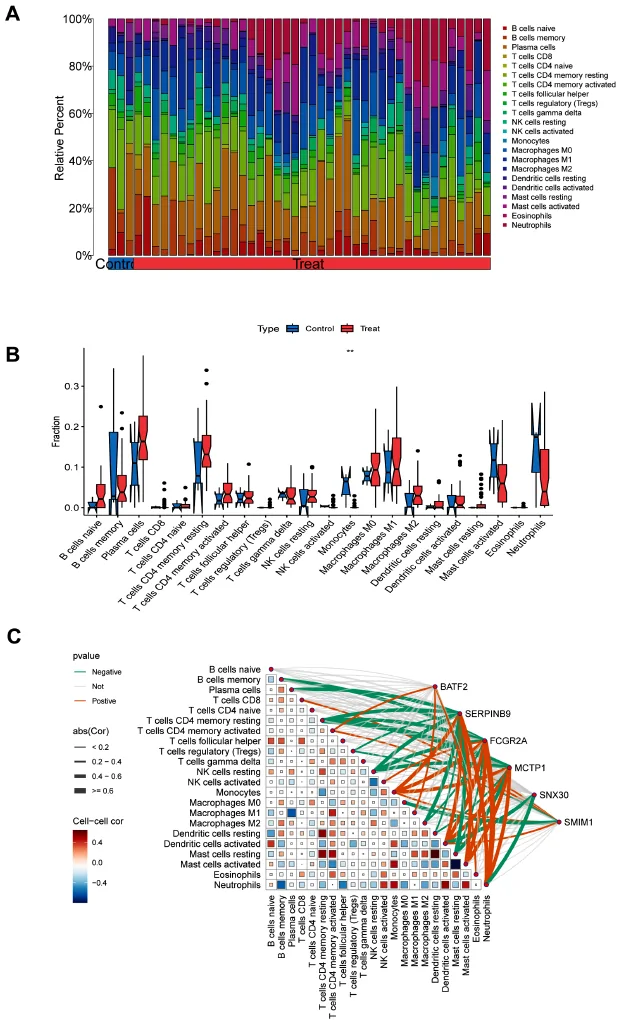
Analysis of immune cell types and gene expression levels in HC and CD groups based on the CIBERSORT algorithm. Blue indicates the healthy control (HC) group, and red indicates the Crohn’s disease (CD) group. (**A**) Stacked bar chart showing the relative proportions of immune cell types. Each bar represents a sample, with different colors indicating different types of immune cells. (**B**) Box plot comparing the scores of immune cell types. *p*<0.05; **p<0.01. (**C**) Cell-gene correlation network. Colors represent the direction and strength of the correlation (red/orange for positive, blue/green for negative), and line thickness indicates the strength of the correlation.

**Fig. (8):**
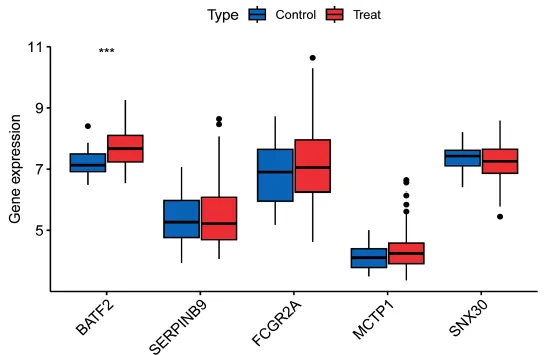
Validation group differential analysis. Blue represents the control group, and red represents the disease group. Asterisks (*) in the figure indicate levels of statistical significance, ***p<0.001.

**Fig. (9):**
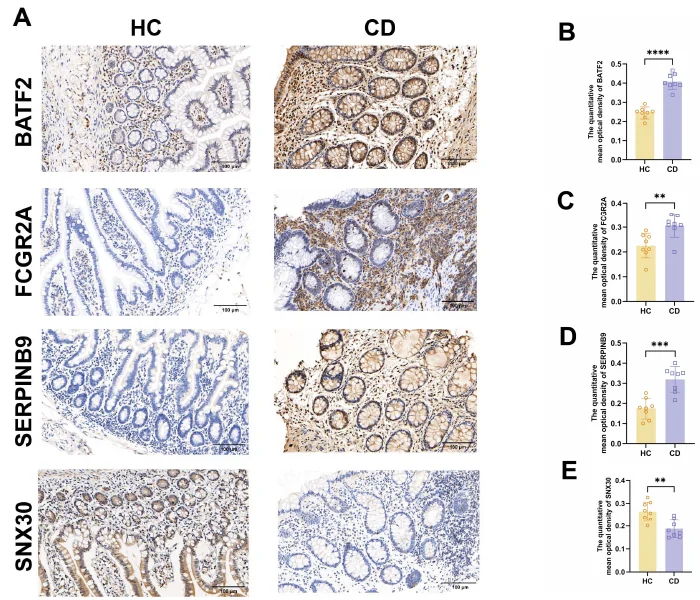
Schematic and statistical diagram of immunohistochemical staining of intestinal tissues in the two groups (n=8). (**A**) Schematic immunohistochemistry of BATF2, FCGR2A, SERPINB9, and SNX30 in the intestinal tissues of the two groups (scale bar= 100 μm). (**B**) Changes in the mean optical density values of the positive signal of BATF2 in the two groups. (**C**) Changes in the mean optical density values of the positive signal of FCGR2A in the two groups. (**D**) Changes in the mean optical density values of the positive signal of SERPINB9 in the two groups. (**E**) Changes in the mean optical density values of the positive signal of SNX30 in the two groups. ** *p*<0.01, *** *p*<0.001, **** *p*<0.0001.

**Table 1:** Dataset information.

GEO Accession	Samples	Sample Source	Platform	Experiment Type	Year
GSE16879	37 cases and 12 controls	Intestinal Biopsy	GPL570	Array	2009
GSE36807	13 cases and 7 controls	Intestinal Biopsy	GPL570	Array	2013
GSE112366	141 cases and 26 controls	Intestinal Biopsy	GPL13158	Array	2019

**Table 2:** Demographics of participants.

-	Health Control (HC)	Crohn's Disease (CD)
Total number	8	8
Female Male Age (year)	2 6 61±14.90	2 6 29.13±6.58
Tissue	Small intestine (partial)	Ileum/Colon

## Data Availability

The original contributions presented in the study are included in the article; further inquiries can be directed to the corresponding authors.

## LIST OF ABBREVIATIONS

CD: Crohn's Disease
MR: Mendelian Randomization
UC: Ulcerative Colitis
GWAS: Genome-wide Association Studies
SNPs: Single-nucleotide Polymorphisms
IVW: Inversevariance Weighting
GSEA: Gene Set Enrichment Analysis
IHC: Immunohistochemistry
eQTL: Expression Quantitative Trait Loci

## STANDARDS OF REPORTING

STROBE-MR guidelines were followed.
